# Polygenic risk of ischemic stroke is associated with cognitive ability

**DOI:** 10.1212/WNL.0000000000002306

**Published:** 2016-02-16

**Authors:** Sarah E. Harris, Rainer Malik, Riccardo Marioni, Archie Campbell, Sudha Seshadri, Bradford B. Worrall, Cathie L.M. Sudlow, Caroline Hayward, Mark E. Bastin, John M. Starr, David J. Porteous, Joanna M. Wardlaw, Ian J. Deary

**Affiliations:** From the Centre for Cognitive Ageing and Cognitive Epidemiology (S.E.H., R. Marioni, C.L.M.S., M.E.B., J.M.S., D.J.P., J.M.W., I.J.D.), Centre for Clinical Brain Sciences (C.L.M.S., M.E.B., J.M.W.), Medical Research Council Human Genetics Unit, Institute of Genetics and Molecular Medicine (C.H.), Alzheimer Scotland Dementia Research Centre (J.M.S.), and Department of Psychology (I.J.D.), University of Edinburgh; Medical Genetics Section (S.E.H., R. Marioni, A.C., C.L.M.S., D.J.P.), University of Edinburgh Centre for Genomic and Experimental Medicine and MRC Institute of Genetics and Molecular Medicine, Western General Hospital, Edinburgh, UK; Institute for Stroke and Dementia Research (R. Malik), Klinikum der Universität München, Ludwig-Maximilians-Universität, Munich, Germany; Queensland Brain Institute (R. Marioni), The University of Queensland, Brisbane, Australia; Department of Neurology (S.S.), Boston University School of Medicine, Framingham; The Framingham Heart Study (S.S.), Framingham, MA; and Departments of Neurology and Public Health Sciences (B.B.W.), University of Virginia, Charlottesville.

## Abstract

**Objectives:**

We investigated the correlation between polygenic risk of ischemic stroke (and its subtypes) and cognitive ability in 3 relatively healthy Scottish cohorts: the Lothian Birth Cohort 1936 (LBC1936), the Lothian Birth Cohort 1921 (LBC1921), and Generation Scotland: Scottish Family Health Study (GS).

**Methods:**

Polygenic risk scores for ischemic stroke were created in LBC1936 (n = 1005), LBC1921 (n = 517), and GS (n = 6,815) using genome-wide association study summary data from the METASTROKE collaboration. We investigated whether the polygenic risk scores correlate with cognitive ability in the 3 cohorts.

**Results:**

In the largest cohort, GS, polygenic risk of all ischemic stroke, small vessel disease stroke, and large vessel disease stroke, but not cardioembolic stroke, were correlated with both fluid and crystallized cognitive abilities. The highest correlation was between a polygenic risk score for all ischemic stroke and general cognitive ability (*r* = −0.070, *p* = 1.95 × 10^−8^). Few correlations were identified in LBC1936 and LBC1921, but a meta-analysis of all 3 cohorts supported the correlation between polygenic risk of ischemic stroke and cognitive ability.

**Conclusions:**

The findings from this study indicate that even in the absence of stroke, being at high polygenic risk of ischemic stroke is associated with lower cognitive ability.

The common cause theory of aging predicts that a proportion of the variance in both age-related cognitive and physical decline is attributable to common biological processes.^1^ Exploring the genetic links between cognitive function and diseases, such as ischemic stroke, may provide insight. Risk factors for cognitive decline and ischemic stroke overlap.^1,2^ Low cognitive ability in youth portends a greater risk of stroke in later life.^3,4^ Cognitive ability in later life is determined by cognitive ability in youth^5^ and rate of cognitive decline.

With the exception of an association between *APOE* and cognitive ability in later life and age-related cognitive decline,^6,7^ few individual genes have reliably been associated with variance in normal cognitive ability. However, Genome-wide Complex Trait Analyses indicates that 29% to 50% of the variance in cognition measured at a single time^8,9^ and about 24% of the variance in lifetime cognitive change^10^ can be attributed to common genetic variants. Heritability, calculated from genome-wide single-nucleotide polymorphism (SNP) data, is estimated at 38% for all ischemic stroke, 40% for large vessel disease (LVD), 33% for cardioembolic (CE) stroke, and 16% for small vessel disease (SVD) stroke.^11^

We used genome-wide association study (GWAS) summary data from the METASTROKE Collaboration^12^ to generate polygenic risk scores for ischemic stroke and its subtypes, SVD, LVD, and CE, in 3 Scottish cohorts consisting of relatively healthy participants—Generation Scotland: the Scottish Family Health Study (GS), Lothian Birth Cohort 1921 (LBC1921), and Lothian Birth Cohort 1936 (LBC1936). We investigated whether the polygenic risk scores predicted cognitive ability/cognitive decline in the 3 cohorts.

## METHODS

### Cohorts.

#### LBC1936.

LBC1936 comprises 1,091 (548 men) relatively healthy older participants, most of whom took part in the Scottish Mental Survey of 1947, when they were about 11 years old.^13^ At a mean age of 69.5 years (SD 0.8), they were enrolled in a study designed to determine factors that influence cognitive aging.^14,15^ They took a number of cognitive and physical tests including the Moray House IQ Test (MHT) No. 12 (a test of general cognitive function), which had been administered at age 11.

Medical history, including history of stroke, was recorded. For this study, a general fluid (gf) cognitive ability score was derived from principal components analysis of 6 Wechsler Adult Intelligence Scale–III UK^16^ nonverbal subtests (Matrix Reasoning, Letter Number Sequencing, Block Design, Symbol Search, Digit Symbol, and Digit Span Backward), as described previously.^17^ We also used MHT scores from age 11 and age 70 years, and the National Adult Reading Test (NART),^18^ taken at age 70 years, as a measure of crystallized cognitive ability. A general cognitive ability score was created as for the gf score plus the addition of NART. Cognitive measures were adjusted for age in days and sex before analysis. To obtain a measure of cognitive aging from age 11 to age 70, gf was corrected for age-adjusted age 11 MHT scores, age, and sex.

#### LBC1921.

LBC1921 comprises 550 (234 men) relatively healthy older participants, most of whom took part in the Scottish Mental Survey of 1932, when they were about 11 years old.^19^ At a mean age of 79.1 years (SD 0.6), they were enrolled in a study designed to determine factors that influence cognitive aging.^5,15^

They took a number of cognitive and physical tests including the MHT, which had been administered at age 11. For this study, a gf cognitive ability score was created from principal components analysis of MHT, Raven Matrices, Logical Memory, and Verbal Fluency, as described previously.^8^ We also used MHT scores from age 11 and age 79 years and the NART^18^ from age 79 years. A general cognitive ability score was created as for the gf score plus the addition of NART. Cognitive measures were adjusted for age in days and sex before analysis. To obtain a measure of cognitive aging from age 11 to age 79, gf was corrected for age-adjusted age 11 MHT scores, age, and sex.

#### Generation Scotland: The Scottish Family Health Study.

GS is a family-structured, population-based cohort study. Between 2006 and 2011, 24,084 participants were recruited in Glasgow, Tayside, Ayrshire, Arran, and North East Scotland. Participants range between 18 and 100 years old and there are up to 4 generations per family.^20,21^

The cognitive tests applied were Logical Memory, Digit Symbol, Verbal Fluency, and Mill Hill Vocabulary. The Logical Memory test is a test of immediate verbal declarative memory from the Wechsler^22^ Memory Scale–III UK. It involves immediate and delayed recall of a story with 25 elements that is read aloud to the participant. The Digit Symbol test is from the Wechsler Adult Intelligence Scale–III UK and measures speed of information processing.^16^ The Verbal Fluency test^23^ measures executive function. The participant has to name as many words as possible beginning with the letters C, F, and L, and is given 1 minute for each letter. The sum of these 3 scores is taken as the overall measure. A gf cognitive ability score was created from principal components analysis of Logical Memory, Digit Symbol, and Verbal Fluency. The Mill Hill Vocabulary test^24^ (Junior and Senior Synonyms combined) was used as a measure of crystallized cognitive ability. A general cognitive ability score was derived as for the gf score plus the addition of the Mill Hill Vocabulary test. Cognitive measures were adjusted for age in years and sex.

Medical history, including history of stroke, was recorded. There were 10,000 white European participants, born in the UK and with near complete phenotype data, selected for genotyping. In the present study, 6,815 unrelated participants (2,813 men), with a mean age of 55.5 years (SD 11.4), were analyzed.

### DNA extraction.

DNA was extracted from blood or buccal cells using standard procedures at MRC Technology Edinburgh (LBC1921) and the Wellcome Trust Clinical Research Facility Genetics Core Edinburgh (LBC1936 and Generation Scotland).

### Standard protocol approvals, registrations, and patient consents.

Ethical approval was attained for LBC1921 and LBC1936 from the Lothian Research Ethics Committee and for LBC1936 from Scotland's Multicentre Research Ethics Committee and for Generation Scotland from the Tayside Research Ethics Committee.

### Creating stroke polygenic risk scores.

DNA samples were genotyped at the Wellcome Trust Clinical Research Facility using the Illumina 610-Quadv1 array (San Diego) (LBC1936 and LBC1921)^8^ or the Illumina HumanOmniExpressExome (San Diego) (Generation Scotland). Individuals were excluded based on relatedness (n = 8 in LBC1936; n = 1 in LBC1921; n = 3,045 in GS), unresolved sex discrepancy (n = 12 in LBC1936; n = 1 in LBC1921; n = 14 in GS), low call rate (≤0.95 n = 16 in LBC1936; n = 5 in LBC1921; ≤0.97 n = 117 in GS), evidence of non-European descent (n = 1 in LBC1936; n = 2 in LBC1921), and pedigree mismatch (n = 8 in GS). SNPs were used in the analyses if they had a call rate ≥0.98, a minor allele frequency ≥0.01, and a Hardy-Weinberg equilibrium test with *p* ≥ 0.001. The first 4 components from a multidimensional scaling analysis of the SNP data were used as covariates in the analyses to control for population stratification.

GWAS summary data for ischemic stroke and its subtypes were obtained from the METASTROKE Consortium.^12^ Summary data included SNP name (rs number) and effect allele and size for imputed SNPs associated with all ischemic, SVD, LVD, or CE stroke subtypes. The information was obtained for 5 different *p* value thresholds: <0.8, <0.5, <0.1, <0.05, and <0.01. The ischemic stroke GWAS included 12,389 cases of all subtypes, SVD 1,894 cases, LVD 2,167 cases, and CE 2,365 cases. The same 62,004 controls were used in each GWAS. Twenty polygenic stroke risk scores (4 stroke phenotypes at 5 *p* value thresholds) were thus created for each participant of LBC1936, LBC1921, and GS as described in Ref. 25. A/T and G/C SNPs, SNPs with a minor allele frequency <0.02, and SNPs not present in METASTROKE were removed from LBC1936, LBC1921, and GS. Pruning was performed to remove those in linkage disequilibrium (*r*^2^ > 0.25 within a 200-SNP sliding window). Risk scores were then created in PLINK^26^ by summing the product of each of the betas obtained from METASTROKE and the number of effect alleles carried by the participant.

### Statistical analyses.

For all cohorts, partial correlations were calculated between the 20 stroke polygenic risk scores and the cognitive phenotypes described above. Covariates included the number of nonmissing SNPs used to form the risk score, and 4 multidimensional scaling components. Where possible (in LBC1936 and GS), individuals with a self-reported history of stroke before the cognitive testing were removed from the cognitive analyses. Random-effects meta-analyses of the gf, general cognitive ability scores, and measures of crystallized cognitive ability (NART in LBC1936 and LBC1921, and Mill Hill Vocabulary in GS) were performed in R (MAc and Metafor packages). An omnibus effect size and standard error were derived and sample heterogeneity was investigated using Cochran *Q* statistic, which calculated the sum of squared deviations of each cohort's effect size from the overall meta-analytic estimate of significance.

## RESULTS

Fifty LBC1936 members (5%) and 127 GS members (1.9%) reported having had a stroke before cognitive testing and were excluded from the analyses. The number of SNPs that made up the polygenic risk scores is shown in table e-1 on the *Neurology*® Web site at Neurology.org.

### LBC1936.

In LBC1936, 7 of 120 correlations between cognitive test scores and polygenic risk scores for ischemic stroke (and its subtypes) reached a significance level of *p* < 0.05 (table 1). All correlations indicated that higher polygenic risk of ischemic stroke (and its subtypes) was associated with higher cognitive ability.

**Table 1:**
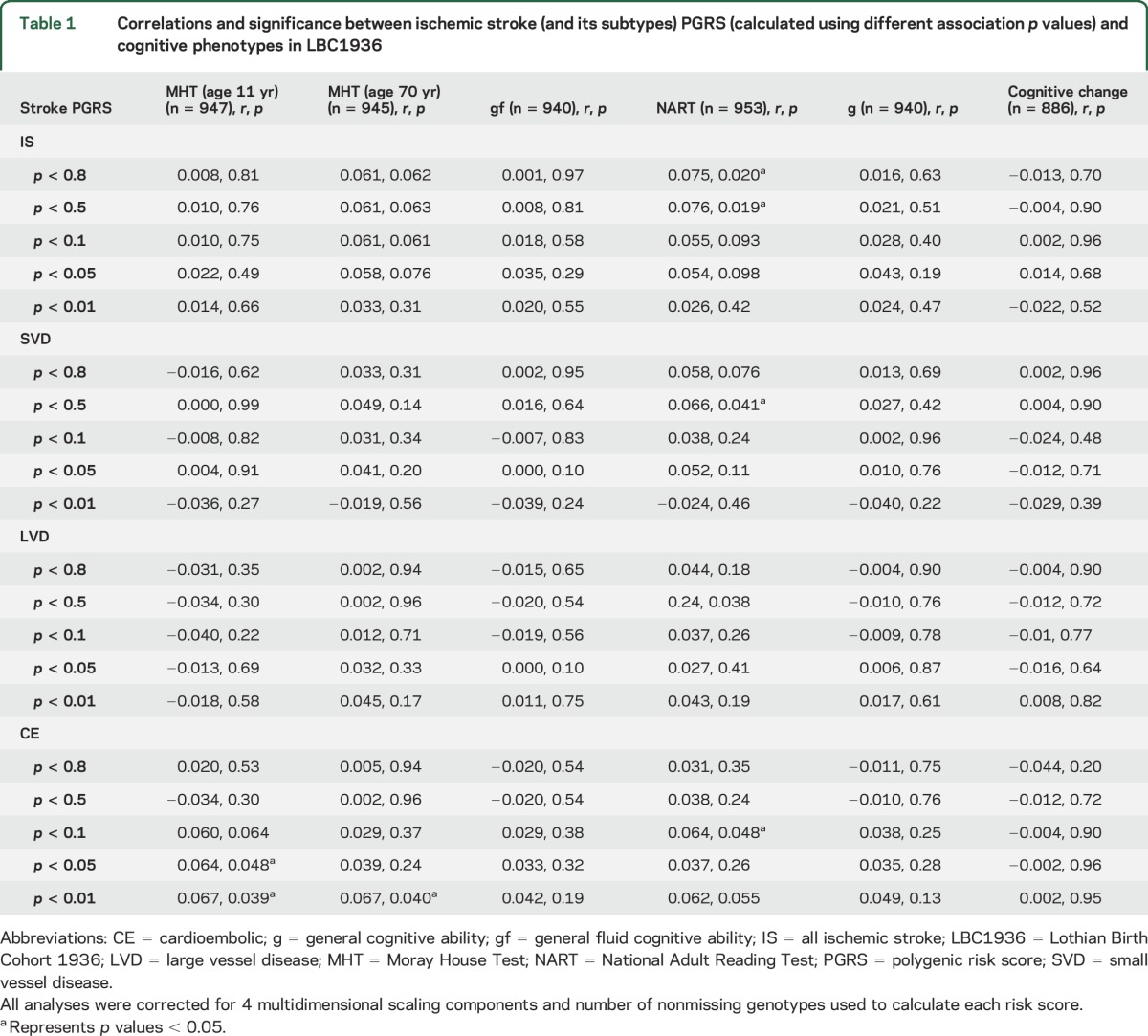
Correlations and significance between ischemic stroke (and its subtypes) PGRS (calculated using different association *p* values) and cognitive phenotypes in LBC1936

### LBC1921.

In the relatively small LBC1921, there is a general trend indicating that higher polygenic risk of ischemic stroke (and its subtypes) is associated with lower cognitive ability, with 4 of 120 reaching a significance level of *p* < 0.05 (table 2).

**Table 2:**
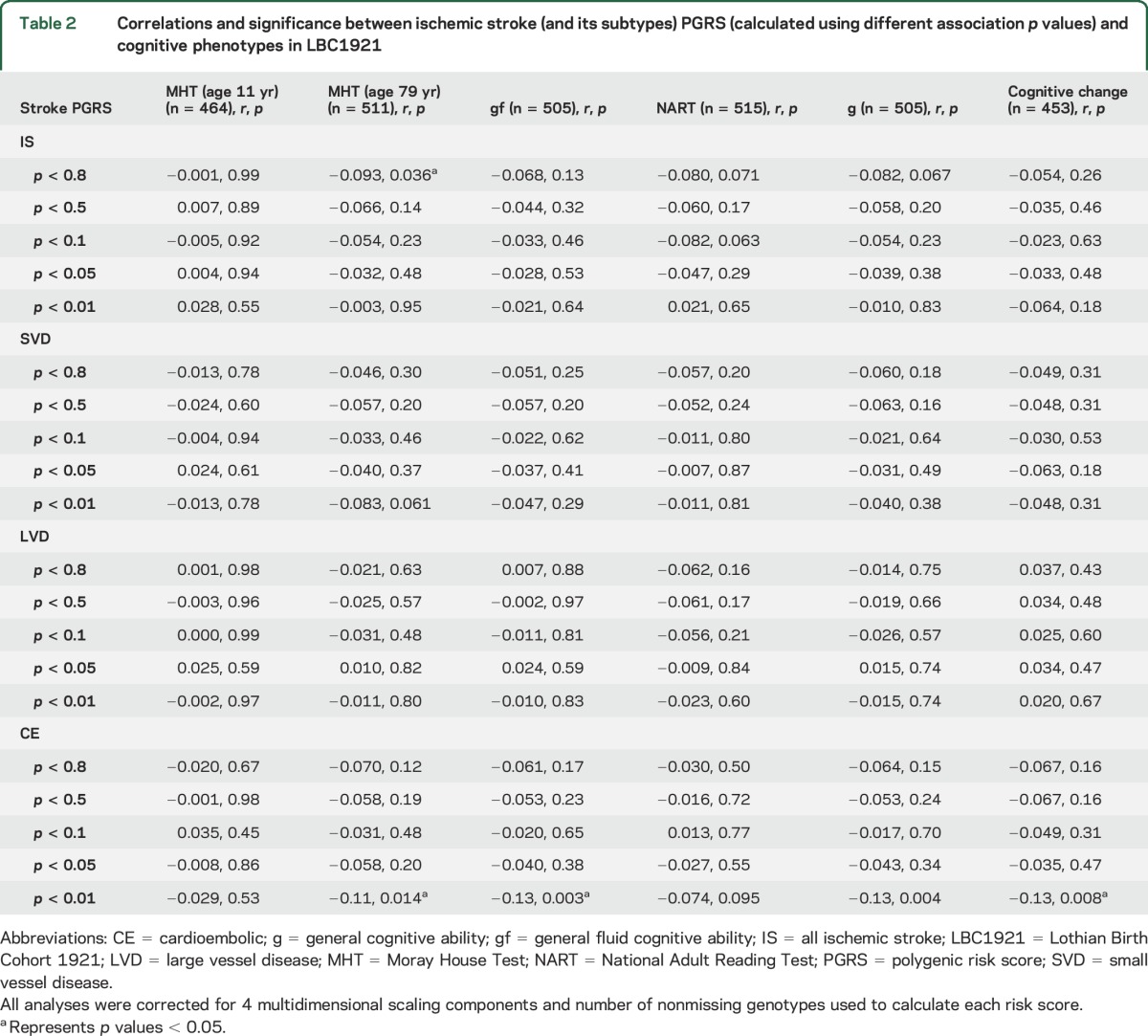
Correlations and significance between ischemic stroke (and its subtypes) PGRS (calculated using different association *p* values) and cognitive phenotypes in LBC1921

### GS.

In GS, 71 of 120 correlations between cognitive test scores and polygenic risk scores for ischemic stroke (and its subtypes) reached a significance level of *p* < 0.05 (table 3). All correlations between polygenic risk of all ischemic stroke, SVD stroke, and LVD stroke and cognitive abilities indicated that higher polygenic risk is associated with lower cognitive abilities. Correlations between polygenic risk of CE stroke and cognitive abilities indicated that higher polygenic risk of CE stroke is associated with higher cognitive abilities.

**Table 3:**
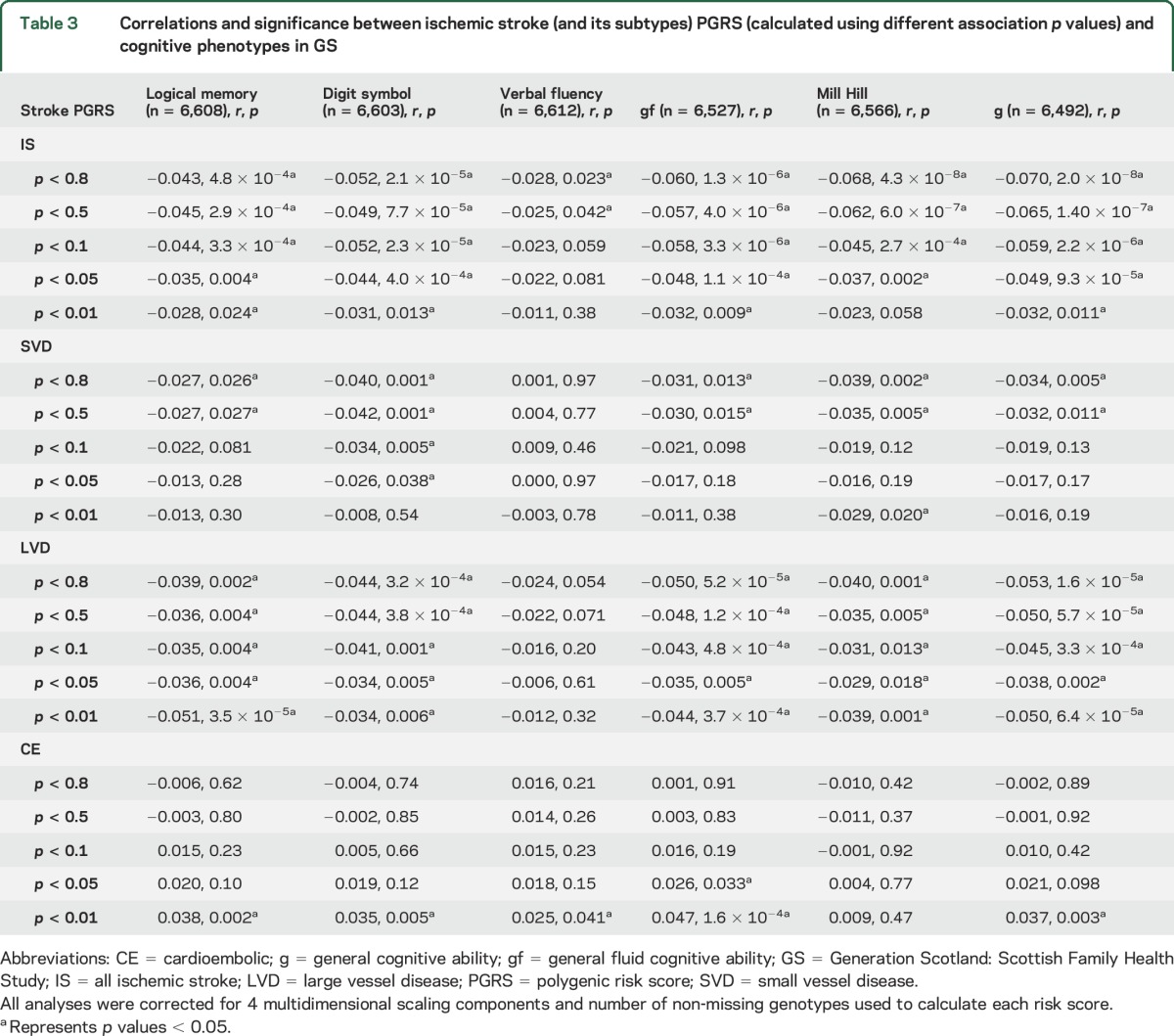
Correlations and significance between ischemic stroke (and its subtypes) PGRS (calculated using different association *p* values) and cognitive phenotypes in GS

### Meta-analysis: Cognition.

In the meta-analysis, 14 of 60 correlations between cognitive test scores and polygenic risk scores for ischemic stroke (and its subtypes) reached a significance level of *p* < 0.05 (table e-2). Sample heterogeneity was indicated for many of the crystallized and general cognitive ability analyses. All correlations between polygenic risk of all ischemic stroke, SVD stroke, and LVD stroke and cognitive abilities indicated that higher polygenic risk is associated with lower cognitive abilities. Correlations between polygenic risk of CE stroke and cognitive abilities indicated that higher polygenic risk of CE stroke is associated with higher cognitive abilities.

## DISCUSSION

In the largest (by 5- and 10-fold over LBC1936 and LBC1921, respectively) of the cohorts (GS), we found an association between higher polygenic risk of all ischemic stroke and lower cognitive ability. Correlations were generally higher for general cognitive ability than the specific tests, suggesting that the greatest influence is on general cognitive ability. The lowest correlations were identified for Verbal Fluency. We found similar correlations between polygenic risk of SVD and LVD stroke and cognitive ability, albeit with smaller effects. For all ischemic and SVD stroke, the polygenic risk scores containing more SNPs generally correlated more strongly indicating that, as with polygenic risk of schizophrenia,^27^ many SNPs with very small effect sizes contribute to these risk scores. High polygenic risk of CE stroke was not associated with low cognition, perhaps because the mechanisms leading to CE stroke may involve blood clotting rather than neurovascular integrity, the former possibly having less of an effect on cognition.

In GS, polygenic risk of all ischemic stroke was a better predictor of cognitive ability than polygenic risk of specific subtypes, and had greater power. We note that the polygenic risk scores for all ischemic stroke were created from a GWAS of >12,000 stroke cases, whereas polygenic risk scores for each of the subtypes were created using data from only approximately 2,000 cases. Also, although METASTROKE used the Trial of Org 10172 in Acute Stroke Treatment (TOAST)^28^ to classify stroke subtypes, this is imprecise, depends on detailed investigations for accurate phenotyping, and therefore some cases may have been assigned incorrectly. Undetected vascular events within METASTROKE participants could also lead to incorrect classification of ischemic stroke subtype.

Correlations between cognitive scores and stroke polygenic risk scores in the relatively small LBC1921 were generally very similar to those in GS but the majority had *p* > 0.05. In LBC1936, the majority of the correlations were in the opposite direction to our hypothesis. It is possible that, as the participants in LBC1936 are all aged about 70 years and still relatively healthy, they may, as a group, have a low polygenic risk of stroke. Only 5% of the cohort reported having experienced a stroke before cognitive testing and these were removed before the analyses. Including the 50 participants who had experienced a stroke made little difference to the results. LBC1921 is also a relatively healthy older cohort. However, incidence of stroke was not available and it is possible that the trend toward a correlation between cognitive ability at age 79 and stroke polygenic risk scores was driven by participants who had experienced cognitive decline following a stroke. Meta-analyses results also indicated that sample heterogeneity was present.

Both fluid and crystallized cognitive ability were associated with polygenic risk of all ischemic, SVD, and LVD stroke in GS. Although fluid cognitive ability tends to decline in later life, crystallized cognitive ability, as measured in this study by tests of vocabulary, remains relatively stable and is therefore a good proxy measure of cognitive ability earlier in life.^29^ A previous study with the LBC1936 cohort indicated that higher cognitive ability measured at age 11 years was associated with fewer white matter hyperintensities at age 73 years.^30^ These data suggest that genetic variants that predispose individuals to risk of ischemic stroke or to risk of brain damage should they have an ischemic trigger in later life, might act much earlier to influence cognitive ability, possibly through influencing brain integrity or brain circulation. It is also possible that certain genes influence cognitive ability through other pathways, for example, developmental pathways, oxidative stress pathways, neurotransmitter pathways,^31^ and, thereafter, individuals with lower cognitive ability might be more likely to lead lifestyles that predispose them to ischemic stroke. Finally, the same genes may be influencing both risk of ischemic stroke and cognitive ability through independent pathways.

One strength of this study is the large population-based cohort of GS. This allowed us to test the hypothesis that high polygenic risk of ischemic stroke is associated with lower cognitive ability even in the absence of stroke. A limitation was that LBC1921 and LBC1936 are relatively small older cohorts. Although the 3 cohorts consist of individuals born in different time periods, we have twin- and family-based and DNA-based evidence that the heritability of cognitive ability is similar over these eras, and the genetic variants influencing cognition will not have changed during this time period.^8,9,32^ However, cognitive ability in later life is an interaction between genetics and environmental factors and each cohort experienced very different situations, which may have had an important influence on cognition, and hence, may have influenced the results. All of the cohorts were composed of largely healthy individuals, so the effect sizes expected were small. A further limitation is that we lacked information on incidental vascular changes in the Scottish cohorts at the time of cognitive testing. The results may have been driven by individuals with undetected stroke. However, MRI data are available for about 700 members of LBC1936 at age 73 years (3 years after the cognitive testing data used in this report were collected). At a mean age of 73 years, as expected for an older population, some white matter hyperintensities were evident in 97% of LBC1936. However, only 11% of the cohort did not self-report having had a stroke, but did have imaging evidence of a stroke.^30^ Population-based studies indicate that white matter hyperintensities increase with age.^33^ Therefore, we expect that the percentage of individuals with undetected stroke and white matter hyperintensities will be lower in LBC1936 at mean age 70 years and lower still in GS, which has a mean age of 55 years, and slightly higher in LBC1921 at mean age 79 years.

We have presented uncorrected *p* values as all cognitive phenotypes are correlated (*r* range: 0.19–0.70, *p* < 0.001; tables e-3–e-5), as are many of the polygenic risk scores (tables e-6–e-8). However, it is possible that some of the findings may be attributable to type 1 error. Future studies on larger groups with more cognitive decline—especially where that is suspected to be of vascular origin—might show larger effect sizes. In the future, it would be helpful to be able to create subtype risk scores based on larger numbers of accurately phenotyped ischemic stroke patients.

The findings from this study indicate that even in the absence of stroke, being at high polygenic risk of ischemic stroke is associated with lower cognitive ability. This may be attributable to a genetic predisposition to defects in brain integrity or circulation, which increases the risk of stroke or reduces resilience to withstand the effect of ischemic triggers on brain damage. Alternatively, the genes may be acting independently to influence cognitive ability and stroke risk through different pathways.

## Supplementary Material

Data Supplement

Coinvestigators

## GLOSSARY

CE: cardioembolic
gf: general fluid
GS: Generation Scotland: Scottish Family Health Study
GWAS: genome-wide association study
LBC1921: Lothian Birth Cohort 1921
LBC1936: Lothian Birth Cohort 1936
LVD: large vessel disease
MHT: Moray House Test
NART: National Adult Reading Test
SNP: single-nucleotide polymorphism
SVD: small vessel disease
