# Supplementary material for: Polygenic risk of ischemic stroke is associated with cognitive ability
Source: Neurology. 2016 Feb 16;86(7):611–8. doi: 10.1212/WNL.0000000000002306 (PMC4762420; doi:10.1212/WNL.0000000000002306)
Supplement: Data Supplement [file supp_86_7_611__index.html]

Data Supplement 

# Polygenic risk of ischemic stroke is associated with cognitive ability

## Data Supplement

Eight tables; one PDF file and one Microsoft Excel file.

**Neurology® data supplements are not copyedited before publication. Published editorials and translations have been copyedited.  
 © 2016 American Academy of Neurology.  
  
 Files in this Data Supplement:**

- Tables e-1 to e-5 - PDF file
- Tables e-6 to e-8 - Microsoft Excel file
