# Supplementary material for: Polygenic risk of ischemic stroke is associated with cognitive ability
Source: Neurology. 2016 Feb 16;86(7):611–8. doi: 10.1212/WNL.0000000000002306 (PMC4762420; doi:10.1212/WNL.0000000000002306)
Supplement: Data Supplement [file supp_WNL.0000000000002306_Tables_e-1-5.pdf]

**Supplemental Tables**

| | **IS** | **SVD** | **LVD** | **CE** |
|---|---|---|---|---|
| P<0.8 | 106,600-109,967 | 107,538-110,887 | 109,847-113,328 | 105,676-109,225 |
| p<0.5 | 63,915-65,631 | 64,240-66,004 | 66,226-68,077 | 63,213-66,582 |
| p<0.1 | 12,329-12,661 | 12,245-12,665 | 12,798-13,220 | 12,062-12,367 |
| p<0.05 | 6,049-6,328 | 6,150-6,336 | 6,407-6,602 | 6,011-6,183 |
| p<0.01 | 1,275-1,321 | 1,224-1,265 | 1,324-1,388 | 1,288-1,314 |

**table e-1. The number of SNPs at each SNP set criteria, across the cohorts, that made up the polygenic risk scores. IS=all ischaemic stroke, SVD=small-vessel disease, LVD=large-vessel disease, CE=cardioembolic.**

| Stroke<br><br>PGRS | | General fluid ability<br><br>(N=7,972) | Crystallised ability<br><br>(N=8,034) | General ability<br><br>(N=7,937) |
|---|---|---|---|---|
| **IS** P<0.8 | r(se),p | **-0.046 (0.020), 0.019** | -0.024 (0.049), 0.62 | -0.046 (0.030), 0.12 |
| | $Q_p$ | 0.20 | **1e-04** | **0.042** |
| **IS** p<0.5 | r(se),p | -0.038 (0.021), 0.079 | -0.016 (0.046), 0.73 | -0.037 (0.028), 0.19 |
| | $Q_p$ | 0.018 | **3e-04** | **0.048** |
| **IS** p<0.1 | r(se),p | -0.031 (0.025), 0.22 | -0.022 (0.039), 0.56 | -0.031 (0.029), 0.28 |
| | $Q_p$ | 0.088 | **0.0084** | **0.044** |
| **IS** p<0.05 | r(se),p | -0.019 (0.027), 0.49 | -0.010 (0.031), 0.73 | -0.018, 0.030, 0.55 |
| | $Q_p$ | 0.057 | **0.028** | **0.031** |
| **IS** p<0.01 | r(se),p | -0.020 (0.017), 0.26 | 0.0047 (0.019), 0.80 | -0.015 (0.019), 0.41 |
| | $Q_p$ | 0.33 | 0.26 | 0.26 |
| **SVD** P<0.8 | r(se),p | **-0.028 (0.011), 0.011** | -0.013 (0.034), 0.71 | **-0.030 (0.011), 0.0090** |
| | $Q_p$ | 0.56 | **0.016** | 0.32 |
| **SVD** p<0.5 | r(se),p | **-0.026 (0.011), 0.020** | -0.0070 (0.036), 0.84 | -0.022 (0.020), 0.27 |
| | $Q_p$ | 0.32 | **0.011** | 0.17 |
| **SVD** p<0.1 | r(se),p | -0.019 (0.011), 0.083 | -0.0045 (0.018), 0.81 | -0.017 (0.011), 0.14 |
| | $Q_p$ | 0.92 | 0.26 | 0.83 |

| | | | | |
|---|---|---|---|---|
| **SVD** p<0.05 | r(se),p | -0.016 (0.011), 0.15 | 0.0043 (0.022), 0.85 | -0.015 (0.011), 0.19 |
| | $Q_p$ | 0.79 | 0.14 | 0.69 |
| **SVD** p<0.01 | r(se),p | -0.017 (0.011), 0.14 | **-0.027 (0.011), 0.015** | -0.020 (0.011), 0.069 |
| | $Q_p$ | 0.56 | 0.92 | 0.71 |
| **LVD** P<0.8 | r(se),p | **-0.036 (0.017), 0.033** | -0.019 (0.030), 0.52 | **-0.036 (0.018), 0.046** |
| | $Q_p$ | 0.31 | **0.030** | 0.29 |
| **LVD** p<0.5 | r(se),p | **-0.042 (0.011), 0.00018** | 0.048 (0.096), 0.61 | **-0.041 (0.014), 0.0029** |
| | $Q_p$ | 0.47 | 0.25 | 0.44 |
| **LVD** p<0.1 | r(se),p | **-0.038 (0.011), 0.00065** | -0.017 (0.024), 0.49 | **-0.040 (0.011), 0.00042** |
| | $Q_p$ | 0.65 | 0.11 | 0.56 |
| **LVD** p<0.05 | r(se),p | -0.020 (0.017), 0.25 | -0.013 (0.019), 0.50 | -0.020 (0.019), 0.29 |
| | $Q_p$ | 0.30 | 0.26 | 0.27 |
| **LVD** p<0.01 | r(se),p | -0.025 (0.019), 0.20 | -0.011 (0.027), 0.69 | -0.024 (0.023), 0.28 |
| | $Q_p$ | 0.24 | 0.06 | 0.13 |
| **CE** P<0.8 | r(se),p | -0.0071 (0.013), 0.58 | -0.0064 (0.011), 0.57 | -0.0070 (0.011), 0.53 |
| | $Q_p$ | 0.36 | 0.43 | 0.41 |
| **CE** p<0.5 | r(se),p | -0.0037 (0.012), 0.75 | -0.0032 (0.014), 0.82 | -0.0054 (0.011), 0.63 |

| | | | | |
|---|---|---|---|---|
| | $Q_p$ | 0.41 | 0.36 | 0.52 |
| **CE** p<0.1 | r(se),p | 0.015 (0.011), 0.17 | 0.019 (0.021), 0.38 | 0.011 (0.011), 0.30 |
| | $Q_p$ | 0.67 | 0.17 | 0.58 |
| **CE** p<0.05 | r(se),p | **0.023 (0.011), 0.043** | 0.0059 (0.011), 0.59 | 0.019 (0.011), 0.10 |
| | $Q_p$ | 0.34 | 0.47 | 0.33 |
| **CE** p<0.01 | r(se),p | **0.043 (0.011), 0.00015** | 0.0047 (0.034), 0.89 | **0.035 (0.011), 0.001** |
| | $Q_p$ | 0.43 | **0.044** | 0.50 |

**table e-2. Correlations and significance of meta-analysis between ischaemic stroke (and its subtypes) polygenic risk scores (PGRS) (calculated using different association p-values) and cognitive phenotypes for LBC1936, LBC1921 and GS cohorts. p-values <0.05 are in bold. IS=all ischaemic stroke, SVD=small-vessel disease, LVD=large-vessel disease, CE=cardioembolic.**

| p | Moray House Test (age 11) | Moray House Test (age 70) | NART |
|---|---|---|---|
| Moray House Test (age 11) | | 0.695, $3.41 \times 10^{-136}$ | 0.687, $4.26 \times 10^{-133}$ |
| Moray House Test (age 70) | 0.695, $3.41 \times 10^{-136}$ | | 0.664, $3.44 \times 10^{-127}$ |
| NART | 0.687, $4.26 \times 10^{-133}$ | 0.664, $3.44 \times 10^{-127}$ | |

**table e-3. Correlation matrix for LBC1936 cognitive phenotypes. Pearson correlations and p-values are shown.**

| Pearson correlation, p | Moray House Test (age 11) | Moray House Test (age 79) | NART |
|---|---|---|---|
| Moray House Test (age 11) | | 0.695, $7.18x10^{-60}$ | 0.669, $2.60x10^{-61}$ |
| Moray House Test (age 79) | 0.695, $7.18x10^{-60}$ | | 0.671, $3.79x10^{-68}$ |
| NART | 0.669, $2.60x10^{-61}$ | 0.671, $3.79x10^{-68}$ | |

**table e-4. Correlation matrix forLBC1921 cognitive phenotypes. Pearson correlations and p-values are shown.**

| Pearson correlation, p | Logical Memory | Digit Symbol | Verbal Fluency | Mill Hill |
|---|---|---|---|---|
| Logical Memory | | 0.242, $1.71x10^{-89}$ | 0.191, $6.83x10^{-56}$ | 0.336, $2.80x10^{-175}$ |
| Digit Symbol | 0.242, $1.71x10^{-89}$ | | 0.349, $2.75x10^{-190}$ | 0.302, $5.31x10^{-140}$ |
| Verbal Fluency | 0.191, $6.83x10^{-56}$ | 0.349, $2.75x10^{-190}$ | | 0.399, $1.45x10^{-252}$ |
| Crystallised ability | 0.336, $2.80x10^{-175}$ | 0.302, $5.31x10^{-140}$ | 0.399, $1.45x10^{-252}$ | |

**table e-5. Correlation matrix for GS cognitive phenotypes. Pearson correlations and p-values are shown.**

**Table legends for tables e-6, e-7 and e-8 (tables in Excel file Harris_stroke_polygenic_risk_tables e-6 to e-8)**

**table e-6. Correlation matrix for LBC1936 ischaemic stroke polygenic risk scores.**

**table e-7. Correlation matrix for LBC1921 ischaemic stroke polygenic risk scores.**

**table e-8. Correlation matrix for GS ischaemic stroke polygenic risk scores.**
