# Supplementary material for: Polygenic risk of ischemic stroke is associated with cognitive ability
Source: Neurology. 2016 Feb 16;86(7):611–8. doi: 10.1212/WNL.0000000000002306 (PMC4762420; doi:10.1212/WNL.0000000000002306)
Supplement: Coinvestigators [file supp_WNL.0000000000002306_Coinvestigators.pdf]

**Members of the METASTROKE consortium**

Matthew Traylor MSc (Stroke and Dementia Research Centre, St George's University of London, London, UK, METASTROKE Site Investigator), Martin Farrall FRCPath (Wellcome Trust Centre for Human Genetics, Department of Cardiovascular Medicine, University of Oxford, Oxford, UK, METASTROKE Site Investigator), Elizabeth G Holliday PhD (Center for Clinical Epidemiology and Biostatistics, School of Medicine and Public Health, University of Newcastle, and Center for Bioinformatics, Biomarker Discovery and Information-Based Medicine, Hunter Medical Research Institute, NSW, Australia, METASTROKE Site Investigator) Jemma C Hopewell PhD (Clinical Trial Service Unit and Epidemiological Studies Unit, University of Oxford, Oxford, UK, METASTROKE Site Investigator), Yu-Ching Cheng PhD (University of Maryland School of Medicine, Department of Medicine, Baltimore, MD, USA, METASTROKE Site Investigator), Myriam Fornage PhD (University of Texas Health Science Center at Houston, Houston, TX, USA, METASTROKE Site Investigator), M Arfan Ikram MD, PhD (Department of Epidemiology, Department of Neurology and Department of Radiology, Erasmus MC University Medical Center, Rotterdam, Netherlands, Netherlands Consortium for Healthy Ageing, Leiden, Netherlands, METASTROKE Site Investigator), Steve Bevan PhD (Stroke and Dementia Research Centre, St George's University of London, London, UK, METASTROKE Site Investigator), Alex P Reiner MD (Division of Public Health Sciences, Fred Hutchinson Cancer Research Center, Seattle, WA, USA, METASTROKE Site Investigator), Braxton D Mitchell PhD (University of Maryland School of Medicine, Department of Medicine, Baltimore, MD, USA, METASTROKE Site Investigator), Robert Clarke MD (Clinical Trial Service Unit and Epidemiological Studies Unit, University of Oxford, Oxford, UK, METASTROKE Site Investigator), Giorgio B Boncoraglio MD (Department of Cereberovascular Disease, Fondazione Istituto di Ricovero e Cura a Carattere Scientifico (IRCCS) Istituto Neurologico Carlo Besta, Milan, Italy, METASTROKE Site Investigator), Pankaj Sharma FRCP(Imperial College Cerebrovascular Research Unit (ICCRU), Imperial College London, London, UK, METASTROKE Site Investigator), Joshua C Bis PhD (Division of Public Health Sciences, Fred Hutchinson Cancer Research Center, Seattle, WA, USA, METASTROKE Site Investigator), Bruce M Psaty PhD (Deapartments of Medicine and Epidemiology, University of Washington, WA, USA, METASTROKE Site Investigator), Peter M Rothwell FRCP (Stroke Prevention Research Unit, Nuffield Department of Clinical Neuroscience, University of Oxford, Oxford, UK, METASTROKE Site Investigator), Jonathan Rosand MD, MSc (Program in Medical and Population Genetics, Broad Institute of Harvard and MIT, Cambridge, MA, USA, Department of Neurology and Center for Human Genetic Research, Massachusetts General Hospital, Boston, MA, USA, METASTROKE Site Investigator), James F Meschia MD (Stroke and Dementia Research Centre, St George's University of London, London, UK, Wellcome Trust Centre for Human Genetics, University of Oxford, Oxford, UK, METASTROKE Site Investigator), Kari Stefansson PhD (deCODE Genetics, Reykjavik, Iceland, Faculty of Medicine, University of Iceland, Reykjavik, Iceland, METASTROKE Site Investigator), Martin Dichgans MD (Institute for Stroke and Dementia Research, Klinikum der Universitát München, Ludwig-Maximilians-Universität, and Munich Cluster for Systems Neurology (SyNergy), Munich, Germany, METASTROKE Site Investigator), Unnur Thorsteinsdottir PhD (deCODE Genetics, Reykjavik, Iceland and Faculty of Medicine, University of Iceland, Reykjavik, Iceland, METASTROKE Site Investigator); Anita L DeStefano PhD (Department of Biostatistics, Boston University School of Public Health, Boston, MA, USA, METASTROKE Site Investigator); Christopher Levi MD (Centre for Translational Neuroscience and Mental Health Research, University of Newcastle, and Hunter Medical Research Institute, New Lambton, NSW, Australia,

METASTROKE Site Investigator);Solveig Gretarsdottir PhD (deCODE Genetics, Reykjavik, Iceland, METASTROKE Site Investigator);Peter Donnelly, FRS (Wellcome Trust Centre for Human Genetics, Oxford, Chair WTCCC2 management committee, member of data and analysis group); Ines Barroso PhD (Welcome Trust Sanger Institute, Cambridge, Deputy chair of WTCCC2 management committee, member of DNA, genotyping, data QC and informatics group); Jenefer M Blackwell PhD (Telethon Institute for Child Health Research, Australia, member of WTCCC2 management committee, member WTCCC2 publications committee); Elvira Bramon MD (UCL, London, member of WTCCC2 management committee); Matthew A Brown FRACP (University of Queensland Diamantina Institute, Brisbane, Australia, member of WTCCC2 management committee, member WTCCC2 publications committee); Juan P Casas MD (LSHTM, London, member of WTCCC2 management committee); Aiden Corvin MD, (Trinity College, Dublin, member of WTCCC2 management committee, member WTCCC2 publications committee); Panos Deloukas PhD (Wellcome Trust Sanger Institute, Cambridge, UK, member of WTCCC2 management committee, member of DNA, genotyping, data QC and informatics group); Audrey Duncanson PhD, (Wellcome Trust Sanger Institute, Cambridge, UK, member of WTCCC2 management committee); Janusz Jankowski MD (Plymouth University, UK, member of WTCCC2 management committee); Hugh S Markus MD, FRCP (University of Cambridge, UK, member of WTCCC2 management committee); Christopher G Mathew PhD, (Kings College London, UK, member of WTCCC2 management committee, Chair WTCCC2 publications committee); Colin NA Palmer PhD (University of Dundee, UK, member of WTCCC2 management committee); Robert Plomin PhD (Kings College London, UK, member of WTCCC2 management committee); Anna Rautanen PhD (Wellcome Trust Centre for Human Genetics, Oxford UK, member of WTCCC2 management committee); Stephen J Sawcer PhD (Univrsity of Cambridge, UK, member of WTCCC2 management committee); Richard C Trembath F Med Sci (Queen Mary University of London, UK, member of WTCCC2 management committee); Ananth C Viswanathan MD, (Moorfields Eye Hospital, London, UK, member of WTCCC2 management committee); Nicholas W Wood MD (University College London, UK, member of WTCCC2 management committee); Chris CA Spencer PhD (Wellcome Trust Centre for Human Genetics, Oxford, UK, member of data and analysis group, member WTCCC2 publications committee); Gavin Band PhD (Wellcome Trust Centre for Human Genetics, Oxford, UK, member of data and analysis group); Celine Bellenguez PhD (Wellcome Trust Centre for Human Genetics, Oxford, UK, member of data and analysis group); Colin Freeman PhD (Wellcome Trust Centre for Human Genetics, Oxford, UK, member of data and analysis group); Garrett Hellenthal PhD (Wellcome Trust Centre for Human Genetics, Oxford, UK, member of data and analysis group); Eleni Giannoulatou PhD (Wellcome Trust Centre for Human Genetics, Oxford, UK, member of data and analysis group); Matti Pirinen PhD (Wellcome Trust Centre for Human Genetics, Oxford, UK, member of data and analysis group); Richard Pearson PhD (Wellcome Trust Centre for Human Genetics, Oxford, UK, member of data and analysis group); Amy Strange PhD (Wellcome Trust Centre for Human Genetics, Oxford, UK, member of data and analysis group); Zhan Su PhD (Wellcome Trust Centre for Human Genetics, Oxford, UK, member of data and analysis group); Damjan Vukcevic PhD (Wellcome Trust Centre for Human Genetics, Oxford, UK, member of data and analysis group); Cordelia Langford PhD (Wellcome Trust Sanger Institute, Cambridge, UK, member of DNA, genotyping, data QC and informatics group); Sarah E Hunt PhD (Wellcome Trust Sanger Institute, Cambridge, UK, member of DNA, genotyping, data QC and informatics group); Sarah Edkins PhD (Wellcome Trust Sanger Institute, Cambridge, UK, member of DNA, genotyping, data QC and informatics group); Rhian Gwilliam PhD (Wellcome Trust Sanger Institute, Cambridge, UK, member of DNA, genotyping, data QC and informatics group); Hannah Blackburn PhD

(Wellcome Trust Sanger Institute, Cambridge, UK, member of DNA, genotyping, data QC and informatics group); Suzannah J Bumpstead PhD (Wellcome Trust Sanger Institute, Cambridge, UK, member of DNA, genotyping, data QC and informatics group); Serge Dronov PhD (Wellcome Trust Sanger Institute, Cambridge, UK, member of DNA, genotyping, data QC and informatics group); Matthew Gillman PhD (Wellcome Trust Sanger Institute, Cambridge, UK, member of DNA, genotyping, data QC and informatics group); Emma Gray PhD (Wellcome Trust Sanger Institute, Cambridge, UK, member of DNA, genotyping, data QC and informatics group); Naomi Hammond PhD (Wellcome Trust Sanger Institute, Cambridge, UK, member of DNA, genotyping, data QC and informatics group); Alagurevathi Jayakumar PhD (Wellcome Trust Sanger Institute, Cambridge, UK, member of DNA, genotyping, data QC and informatics group); Owen T McCann PhD (Wellcome Trust Sanger Institute, Cambridge, UK, member of DNA, genotyping, data QC and informatics group); Jennifer Liddle PhD (Wellcome Trust Sanger Institute, Cambridge, UK, member of DNA, genotyping, data QC and informatics group); Simon C Potter PhD (Wellcome Trust Sanger Institute, Cambridge, UK, member of DNA, genotyping, data QC and informatics group); Radhi Ravindrarajah PhD (Wellcome Trust Sanger Institute, Cambridge, UK, member of DNA, genotyping, data QC and informatics group); Michelle Ricketts PhD (Wellcome Trust Sanger Institute, Cambridge, UK, member of DNA, genotyping, data QC and informatics group); Matthew Waller PhD (Wellcome Trust Sanger Institute, Cambridge, UK, member of DNA, genotyping, data QC and informatics group); Paul Weston PhD (Wellcome Trust Sanger Institute, Cambridge, UK, member of DNA, genotyping, data QC and informatics group); Sara Widaa PhD (Wellcome Trust Sanger Institute, Cambridge, UK, member of DNA, genotyping, data QC and informatics group); Pamela Whittaker PhD (Wellcome Trust Sanger Institute, Cambridge, UK, member of DNA, genotyping, data QC and informatics group)
